# Self-Compassion and Rumination Type Mediate the Relation between Mindfulness and Parental Burnout

**DOI:** 10.3390/ijerph18168811

**Published:** 2021-08-20

**Authors:** Marine Paucsik, Agata Urbanowicz, Christophe Leys, Ilios Kotsou, Céline Baeyens, Rebecca Shankland

**Affiliations:** 1 University Grenoble Alpes, LIP/PC2S, 38000 Grenoble, France; agata.urbanowicz@univ-grenoble-alpes.fr (A.U.); celine.baeyens@univ-grenoble-alpes.fr (C.B.); 2 Unité de Psychologie Sociale et Interculturelle, University Libre de Bruxelles, 1050 Brussels, Belgium; christophe.leys@ulb.be (C.L.); ilios.kotsou@ulb.ac.be (I.K.); 3 Chair of Economic Peace, Mindfulness, and Well-Being at Work, Grenoble Ecole de Management, 38000 Grenoble, France; 4 Laboratoire DIPHE, University Lumière Lyon 2, 69676 Bron, France

**Keywords:** lockdown, mindfulness, parental burnout, ruminations, self-compassion

## Abstract

The COVID-19 lockdown increased the day-to-day challenges faced by parents, and thereby may have increased parental burnout risk. Therefore, identifying parental burnout protection factors is essential. This study aimed to assess the protective role of the following factors which can be increased through mindfulness practice: trait mindfulness, self-compassion, and concrete vs. abstract ruminations. A total of 459 parents (*M*_age_ = 40; 98.7% female) completed self-reported questionnaires at two-time points to assess the predictive role of mindfulness on parental burnout, self-compassion and rumination type, and the mediating role of self-compassion and rumination type in the relation between mindfulness and parental burnout. Results showed that trait mindfulness, self-compassion, and rumination type at Time 1 predicted levels of parental burnout at Time 2. Self-compassion (indirect effects: b = − 22, 95% CI = [−38, −05], *p* < 0.01), concrete ruminations (indirect effects: b = −20, 95% CI = [−32, −09], *p* < 0.001), and abstract ruminations (indirect effects: b = −0.54, 95% CI = [−71, −37], *p* < 0.001) partially mediated the relation between trait-mindfulness and parental burnout. These findings showed that trait mindfulness, self-compassion, and concrete (vs. abstract) ruminations may help prevent parental burnout in the context of the COVID-19 pandemic. These results contribute to the field of research on parental burnout prevention and will allow for the development of effective approaches to mental health promotion in parents.

## 1. Introduction

The lockdown related to the COVID-19 pandemic has been shown to be a challenging and anxiety-provoking period for the general population [1,2,3]. This situation may have been even more challenging for parents and may have represented a risk factor for parental burnout for parents locked down with children. An increasing number of research studies have focused on what is now called parental burnout, not only in specific parenting situations, but also in the general population [4,5,6]. Parents can develop burnout if they don’t have the resources to deal with the daily challenges of parenting [5,7]. More specifically, parental burnout has been conceptualized as having four dimensions: exhaustion in one’s parenting role, contrast with previous parental self, feelings of being fed up with one’s parenting role, and emotional distancing from one’s children [5]. Parental burnout is thus a multidimensional syndrome which occurs during prolonged exposure to a stressful situation [8]. The period of the COVID-19 pandemic can be considered as a prolonged stressful situation due to social and physical isolation, risk of unemployment, financial pressure, and difficulties balancing work and family life while schools were closed, and is, therefore, likely to increase parental burnout risks [9,10,11]. In this context, the question of the predictive and protective factors of parental burnout appears to be essential. For instance, research has shown that lack of balance between parenting strains and resources could result in parental burnout [12]. In that sense, identification of protective factors could substantially contribute to the prevention and reduction of parental burnout by decreasing the deleterious effects of stress during the lockdown.

Numerous studies have shown that mindfulness meditation effectively improves emotion regulation and reduces stress, anxiety, and depressive symptoms [13]. Mindfulness refers to paying attention to the experience of the present moment and developing a non-judgmental acceptance of it. Thus, mindfulness involves learning to be aware of cognitive and physical signals of high stress levels and to break the detrimental downward spiral that has been identified in past research [14,15,16]. This ability can be learned through formal practices [17,18] which are usually found in mindfulness meditation protocols such as Mindfulness Based Cognitive Therapy (MBCT, [19]) or Mindfulness Based Stress Reduction (MBSR, [20]). However, recent research shows that mindfulness as a trait can also be developed through informal practices [21] facilitating its integration in everyday life. Mindfulness practices can also develop mindfulness as a trait [22]. Thus, it seems important to assess mindfulness as a trait in order to have an accurate measure of this skill. Trait mindfulness has been shown to be effective in reducing anxiety and depression symptoms in the context of the COVID-19 pandemic [23,24]. It is also likely to represent an effective protection factor against parental burnout as shown in Anclair et al.’s study in 2018 [25], which evaluated the impact of mindfulness intervention on parental burnout. Indeed, trait mindfulness has been shown to contribute to greater mental health, resilience, and other essential qualities for parenting such as empathy and parenting self-efficacy [26]. Thus, mindfulness appears to play an important role in parenting although other studies have shown its importance in the quality of romantic and family relationships [27,28]. Trait mindfulness has also been shown to reduce ruminations associated with states of discomfort [29], and to develop self-compassion which is associated with greater mental health and resilience [26,30,31,32]. Indeed, many studies have shown the mediating role of self-compassion and rumination between mindfulness and mental health [33,34]. Self-compassion is described as the ability to address oneself with kindness, warmth, and benevolence [35] and has been shown to reduce anxiety and depression symptoms [36]. In addition, self-compassion helps to reduce self-criticism and perfectionism, dimensions involved in parental burnout [37]. Therefore, self-compassion may play a protective role against parental burnout [12]. Conversely, research has shown that ruminations increase anxiety and depression symptoms [38,39]. Evidence suggests that the type of ruminations (abstract or concrete) has a different impact on anxiety and depression symptoms [40]: abstract ruminations contribute to unconstructive, indistinct, and unclear negative repetitive thoughts, while concrete ruminations represent constructive thoughts focused on one’s experience of the present moment, and are distinct, specific to a situation, unequivocal, clear, and singular [40]. Concrete ruminations are therefore considered to be helpful to cope with difficulties [41]. Thus, the type of rumination, abstract or concrete, is likely to mediate the relationship negatively and positively between mindfulness and parental burnout. Finally, self-compassion and ruminations are two processes associated with respectively lower and higher levels of perfectionism, which is a risk factor for parental burnout [37,42].

Based on the results of previous studies showing the role of mindfulness on parental stress [43] and the processes at play in mindfulness [34], the present study aimed to assess the protective role of trait mindfulness and mindfulness practice against parental burnout during the lockdown period, and the mediating role of the following psychological process: self-compassion, concrete ruminations, and abstract ruminations. In order to test this mediation model based on the results of past studies [34,43,44], we made several assumptions. Before testing our main hypotheses, we wanted to perform a preliminary analysis to verify that mindfulness practice enhances trait mindfulness at T1 and T2. The first and main hypothesis was that trait mindfulness at Time 1 would predict lower parental burnout scores at Time 2. The second hypothesis stated that trait mindfulness would predict higher levels of self-compassion and concrete ruminations, and lower levels of abstract ruminations at Time 2. The third hypothesis stated that self-compassion and concrete ruminations would negatively predict parental burnout, and that abstract ruminations would positively predict parental burnout. Finally, the fourth hypothesis was that the relationship between mindfulness and parental burnout would be mediated in parallel by increased self-compassion and concrete ruminations and by decreased abstract ruminations. By testing these hypotheses, this study will contribute to recent research on parental burnout in order to specify the risk and protective factors. The identification of these factors will also allow the implementation of preventive interventions for parents.

## 2. Materials and Methods

### 2.1. Participants

The longitudinal design of the current study involved the completion of a survey at two-time points separated by one month (May and June 2020). To obtain an effect for multiple linear regression and mediation analyses with a medium effect size (0.25) and acceptable power (i.e., 0.8; with alpha set at 0.008 with Bonferroni corrections), the calculated sample size required was 408. Sensitivity analyses ran on G*Power [45] indicated that with these parameters, it would be possible to detect effect sizes of d 0.23 if they existed. A total of 1202 participants were recruited via different websites for parents from 1–15 May 2020. Eligibility criteria required participants to be: (1) aged 18 years or older; (2) fluent in French; and (3) locked down with at least one child below the age of 18, and (4) certify having read the informed consent and accepted to take part in the study. A total of 743 responses were discarded as they were incomplete.

### 2.2. Procedure

This study was approved by the ethical committee of the local university which issued favorable approval number: CER Grenoble Alpes-Avis-2020-05-01-01. Participants were recruited through an announcement posted on parenting websites. After having read the information sheet and accepted the informed consent, all participants were assigned with a unique identification number allowing them access to an online questionnaire. The informed consent allowed participants to withdraw at any time without having to justify their withdrawal. They were also assured that the data would remain anonymous. To avoid selection bias, participants were not informed that the study focused on parental burnout. The study was presented as a study on “psychological protective factors during the lockdown”. At the end of the first questionnaire, participants could enter their email address on a separate link disconnected from the questionnaire in order to be re-contacted one month later for the second measure. The second evaluation consisted of the same measures as the first evaluation. Participants did not receive any financial compensation for their participation. The online questionnaire required the answers to all questions to limit missing data.

### 2.3. Measures

The online questionnaire consisted of sociodemographic questions and validated scales measuring parental burnout, trait mindfulness, self-compassion, compassion, and rumination type.

Sociodemographic variables: Participants were asked their age, gender, level of education, family and work situations during the lockdown, the number of children and the number of children living with them during the lockdown, and the age of the children. We also asked the participants to report their time of mindfulness practice per week, indicating whether they practice: never, once or twice a week, or several times a week.

Parental Burnout: Parental burnout was assessed using the French version of the Parental Burnout Assessment (PBA) [7]. PBA is a self-reported questionnaire consisting of four subscales: (a) emotional and physical exhaustion in one’s parental role (9 items); (b) contrast with former parental self (i.e., the feeling of not being a good parent anymore; 6 items); (c) feelings of being fed up in one’s parental role (5 items); and (d) emotional distancing from one’s child (3 items). To illustrate the items were e.g., I feel tired when I get up in the morning and have to face another day with my children (exhaustion in the parental role); I feel as though I’ve lost my direction as a dad/mum (contrast in parental self); I can’t stand my role as a father/mother any more (feeling of being fed up); and I do what I’m supposed to do for my child(ren), but nothing more (emotional distancing from the child). The questionnaire consisted of 22 items evaluated on a 7-point Likert scale ranging from: never (0), a few times a year or less (1), once a month or less (2), a few times a month (3), once a week (4), a few times a week (5), or daily (6). The overall parental burnout score was obtained by adding the scores for each dimension. Higher scores indicate higher frequency of parental burnout symptoms. PBA has particularly good psychometric properties [5]. In the current study, Cronbach’s alpha was excellent across the entire scale (α = 0.98), with α = 0.96 for exhaustion in one’s parental role, α = 0.94 for the contrast in parental self, α = 0.95 for the feeling of being fed up in one’s parental role, and α = 0.83 for emotional distancing from the child.

Mindfulness: Mindfulness was assessed using the French version of Mindful Attention and Awareness Scale (MAAS) which measures the frequency of mindful states in day-to-day life [46]. The scale was designed to measure mindfulness as attention focused on the present moment and awareness in everyday experience. The scale consisted of 15 items (e.g., I break or spill things because of carelessness, not paying attention, or thinking of something else; I rush through activities without being really attentive to them). The MAAS is considered applicable regardless of an individual’s personal experience of meditation [47]. The MAAS uses both general and situation-specific statements to assess mindfulness and is scored on a 6-point Likert scale rating from 1 (almost always) to 6 (almost never). After reversing the items, a global mean score is calculated. In the present study, the scale demonstrated good internal reliability with a Cronbach’s alpha of 0.86.

Self-compassion: Self-compassion was measured with the French version of the Self-Compassion Short Form Scale (SCS-SF) [48]. SCS-SF is a 12-item instrument with six subscales assessing elements of self-compassion (i.e., self-kindness, self-judgment, common humanity, isolation, mindfulness, and overidentification). Self-kindness refers to the tendency to extend kindness and understanding toward oneself when feeling emotional pain or stress (e.g., I try to be understanding and patient towards those aspects of my personality I don’t like). Self-judgment reflects the tendency to be self-critical, disapproving, and intolerant toward one’s own flaws and difficult experiences (e.g., I’m disapproving and judgmental about my own flaws and inadequacies). Common humanity is a capacity of recognition that feelings of inadequacy, emotional pain, and failure are universal human experiences (e.g., I try to see my failings as part of the human condition). Isolation dimension measures feelings of loneliness, separation, and disconnection from others at times of failure or distress (e.g., When I’m feeling down, I tend to feel like most other people are probably happier than I am). Mindfulness refers to holding negative thoughts and emotions in balanced awareness, with an open and accepting stance toward difficult feelings and situations (e.g., When something painful happens I try to take a balanced view of the situation). Overidentification refers to the tendency to become excessively immersed in or consumed by negative feelings (e.g., When I fail at something important to me, I become consumed by feelings of inadequacy). Experienced meditators have been shown to score higher than nonmeditators [48]. As the Self-Compassion scale has a partial overlap with the MAAS, we conducted our analyses only on the four sub-dimensions of the SCS-SF: self-kindness, self-judgment, common humanity and isolation. In this study, the scale reliability was good with a Cronbach’s alpha of 0.87 for the total scale and of 0.82 for the two subscales kindness and common humanity.

Rumination type: Abstract and concrete ruminations were assessed using the French version of the Mini-Cambridge-Exeter Repetitive Thinking Scale (Mini-CERTS). This scale was developed and validated by Barnard and collaborators [49] and measures two dimensions of repetitive thinking: “abstract, analytical thinking” (AAT; e.g., My thinking tends to get stuck in a rut, involving only a few themes) and “concrete, experiential thinking” (CET; e.g., I can grasp and respond to changes in the world around me without having to analyze the details). The Mini-CERTS is a 16-items instruments scored on a 4-point Likert scale varying from 1 (almost never) to 4 (almost always). The French version of the questionnaire was validated [41]. In the present study, the scale presented good internal reliability respectively for the abstract ruminations dimension (α = 0.76), and the concrete ruminations dimension (α = 0.79).

### 2.4. Data Analyses

Data were analyzed using JAMOVI Version 1.2.27 (The Jamovi Project, Sidney, Australia) [50]. Correlations between the variables involved in the mediation analyses were computed. Pearson’s r correlations were used for continuous variables and ANOVAs were used for categorical variables.

Missing values analysis found that 58.8% of data was missing at T2 (ranging from 56 to 61.6% missingness at the variable level). A total of 743 individuals failed to complete the second measurement. Little’s MCAR test yielded non-significance, [Little’s MCAR test χ^2^ (618) = 661, *p* = 0.11], indicating that the missing data were missing completely at random. Analyses were therefore performed using only complete available data (N = 459).

Before testing our main hypotheses, we carried out a preliminary analysis to check whether mindfulness practice was significantly related to trait mindfulness scores. To evaluate the practice of mindfulness, participants were asked to rate their mindfulness practice on a scale ranging from: 0 (never, no practice), 1 (sometimes, 1–2 times a week), or 3 (often, more than 3 times a week or several times a day). A two factorial analysis of variance (ANOVA) was performed to test the difference in trait mindfulness scores depending on the time spent practicing mindfulness per week.

Our preliminary analysis of whether mindfulness practice enhance trait mindfulness at T1 and T2 was tested with two repeated measures ANOVA.

To test the first hypothesis according to which trait mindfulness at T1 would predict lower levels of parental burnout at T2, a linear regression analysis was performed assessing the relation between mindfulness at T1 and parental burnout at T2.

To test the second hypothesis stating that trait mindfulness at T1 would predict higher levels of self-compassion and concrete rumination and lower levels of abstract ruminations at T2, several linear regression analyses were performed. We used the same analyses to test the hypothesis stating that self-compassion and concrete ruminations would negatively predict parental burnout, and that abstract ruminations would positively predict parental burnout.

Finally, to test the fourth hypothesis, which postulates that the relationship between mindfulness and parental burnout would be mediated in parallel by the decrease of abstract ruminations and the increase of concrete ruminations and self-compassion, we applied a parallel mediation model with causal step method described by Baron and Kenny (1986), with mindfulness as the independent variable, parental burnout as the dependent variable, and self-compassion and abstract and concrete ruminations as parallel mediating variables.

## 3. Results

### 3.1. Sample Characteristics

The final sample who had responded to both questionnaires consisted of 459 participants, with a majority of women (98.7%), aged between 25 and 59 (M = 40, SD = 6.75). Among the participants, 104 of the parents were single (22.3%) and 355 were couples (77.4%). Of these, 138 had one child at home during COVID-19, 234 participants had two, 65 had three, 14 had four and eight had five or more children. One participant had a Junior high school certificate (0.2%), 13 had a vocational certificate (2.8%), 54 had a high school degree (11.8), 162 had a bachelor’s degree (35.3%), 131 had a master’s degree (28.5), and 98 had a postgraduate degree (21.4%). Most of the participants were working, 173 were doing their work at home (48.8%) and 102 were working at their workplace (28.7%). During the study, 80 participants (22.5%) did not work. Finally, 158 participants had no practice of mindfulness meditation (34.4%), 206 practiced occasionally (44.8%), and 95 practiced regularly (20.8%).

### 3.2. Descriptive Statistics at Time 1

Descriptive statistics for trait mindfulness (MAAS), parental burnout (PBA), self-compassion (SCS), and abstract and concrete ruminations (MINI-A and MINI-C) are presented in Table 1. All the variables were consistently correlated in the expected directions: trait mindfulness was negatively correlated with parental burnout and abstract ruminations, and positively correlated with self-compassion and concrete ruminations. Self-compassion was also negatively correlated with parental burnout and abstract ruminations, and positively correlated with trait mindfulness and concrete ruminations. Abstract ruminations were positively correlated with parental burnout and negatively correlated with concrete ruminations, self-compassion, and trait mindfulness. Concrete ruminations were negatively correlated with parental burnout and abstract ruminations and positively correlated with trait mindfulness and self-compassion.

The average score for mindfulness was in the middle range and the average parental burnout score was higher than in a reference sample; more precisely, it corresponded to a low risk of burnout [7]. The average score for self-compassion was lower than the average score observed in the general population [51], and the average scores for both concrete and abstract ruminations were in the middle range.

### 3.3. Descriptive Statistics at Time 2

Correlation analyses at Time 2 showed similar correlations to those at Time 1 (see Table 2). Trait mindfulness was negatively correlated with parental burnout and abstract ruminations and positively correlated with self-compassion and concrete ruminations. Self-compassion was also negatively correlated with parental burnout and abstract ruminations and positively correlated with trait mindfulness and concrete ruminations. Abstract ruminations were negatively correlated with concrete ruminations, self-compassion, and trait mindfulness and positively correlated with parental burnout. Concrete ruminations were positively correlated with trait mindfulness and self-compassion and negatively correlated with parental burnout and abstract ruminations.

### 3.4. Mindfulness Practice Effect on Trait Mindfulness at T1 and T2

Two repeated measures ANOVAs were conducted to analyze the mean group differences of trait mindfulness according to the three levels of practice: never, sometimes, and often. We observed a main effect of the level of practice on trait mindfulness at T1, F(2, 459) = 7.72, *p* < 0.001, η^2^*_p_* = 0.03, and at T2, F(2, 459) = 12.6, *p* < 0.001, η^2^*_p_* = 0.05.

### 3.5. The Role of Trait Mindfulness on Parental Burnout, Self-Compassion and Rumination Type

The relation between trait mindfulness, parental burnout, self-compassion, and abstract and concrete ruminations over time were analyzed using multiple linear regression analyses. Results showed that mindfulness significantly predicted parental burnout, F(1, 457) = 85.1, *p* < 0.001, with a R2 of 0.16, self-compassion, F(1, 457) = 140, *p* < 0.001, with a R2 of 0.23, abstract ruminations, F(1, 457) = 96.2, *p* < 0.001, with a R2 of 0.17, and concrete ruminations, F(1, 457) = 62.4, *p* < 0.001, with a R2 of 0.12.

### 3.6. The Role of Self-Compassion and Rumination Type on Parental Burnout

The results of linear regression analysis showed a significant and negative relation of self-compassion at T1 and parental burnout at T2, F(1, 457) = 106, *p* < 0.001, with an R2 of 0.19, a significant and positive relation between abstract ruminations at T1 and parental burnout T2, F(1, 457) = 177, *p* < 0.001, with an R2 of 0.28 and a significant and negative relation of concrete ruminations at T1 and parental burnout at T2, F(1, 457) = 92.8, *p* < 0.001, with an R2 of 0.17.

### 3.7. The Mediating Role of Self-Compassion and Rumination Type on the Relationship between Trait-Mindfulness and Parental Burnout

**Figure 1:**
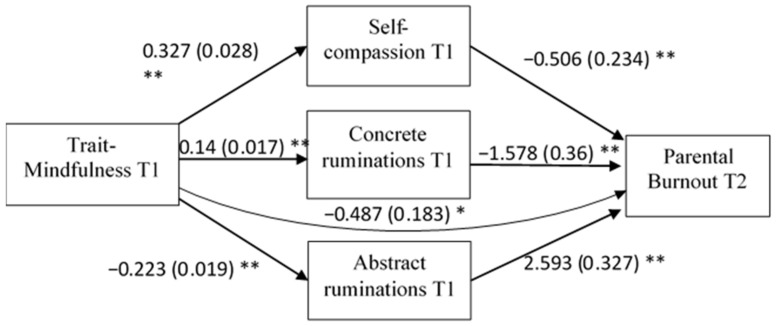
The self-compassion and rumination type mediation model of the relationship between mindfulness and parental burnout. Note: N = 459. Standardized regression coefficients for the relationship between trait-mindfulness, self-compassion, concrete ruminations, abstract ruminations, and pa-rental burnout. Standard errors are in brackets. * *p* < 0.01, ** *p* < 0.001. The data were analysed with Jamovi.

As the variables were significantly correlated in the expected directions, we finally tested our fourth hypothesis regarding the mediation model. The results are presented in Figure 1. The mediation analysis showed that the effect of trait mindfulness on parental burnout was partially mediated by self-compassion, abstract ruminations, and concrete ruminations. The analysis showed the following indirect effects: MAAS T1 × MINI-C T1 × PBA T2 = −0.22 (0.058), *p* < 0.001, MAAS T1 × MINI-A T1 × PBA T2 = −0.570 (0.089), *p* < 0.001, MAAS T1 × SCS T1 × PBA T2 = −0.165 (0.089), *p* < 0.05.

## 4. Discussion

The main objective of this study was to test the predictive and protective role of mindfulness, self-compassion, and concrete ruminations on parental burnout during the COVID-19 lockdown. Consistent with previous research, the results were in line with our first hypothesis: mindfulness was negatively related to parental burnout [25]. These findings are consistent with a growing literature on the relation between mindfulness and parental burnout [26,52,53,54]. Indeed, mindfulness has been associated with lower levels of stress, anxiety, and depression among parents [43,55], with higher levels of emotion regulation [56], more effective conflict management [57], and, more generally, with better family relationships [58,59]. Furthermore, in accordance with our second hypothesis, mindfulness was shown to increase self-compassion [60] and decrease dysfunctional ruminations [61]. As tested with our third hypothesis, these factors appear to impact parental burnout. While self-compassion and concrete ruminations seem to prevent it, abstract ruminations seem to be a risk factor. Mindfulness thus seems to influence several psychological processes [34] that have an impact on parental burnout that we were able to test through our fourth hypothesis with our mediation model. The mediation model showed that the effect of trait-mindfulness was partially mediated by self-compassion and rumination type. The results also showed that these two mediating processes did not fully explain the effect of mindfulness on parental burnout. Indeed, it seems that mindfulness involves other factors that may have an effect on parental burnout, such as acceptance (i.e., a non-judging/non-attached view of experiences), or emotion regulation, and other dimensions identified as mediators of mindfulness [37,62,63,64], which have been identified as protective factors for parental burnout [37,65]. Some limitations must be considered. First, the sample was composed of over 98% women, which limits the external validity of the study. Further research on a population of fathers is needed to generalize these findings. This is a problem frequently encountered in studies on parental burnout. Therefore, future research should be vigilant on this aspect [7,66]. Second, the assessment was based only on self-reported questionnaires, which may have biased the results due to a response bias related to social expectations. Third, prior to the analysis of our hypotheses, we had tested the effect of having participated in a mindfulness program before the lockdown on parental burnout, self-compassion, and ruminations. However, the results did not show significant differences between those who had followed a program and those who had not. This may imply that individuals who had already followed a program may not have practiced sufficiently yet to make a difference compared to other parents. Future studies should evaluate more specifically the impact of mindfulness practices on these different variables.

Finally, research on the evaluation of the protective factors of parental burnout should be continued in order to complete the model which was tested in this research, and which could be completed in the future by the assessment of acceptance, which seems to play a major role in the effectiveness of mindfulness as well as in parental well-being.

The identification of these protection factors will help develop effective interventions specific to the needs of parents. Indeed, currently few interventions have been validated in the field of parental burnout prevention [67]. The results of this study could inform the creation of interventions targeting risk factors such as abstract ruminations, which contribute to parental perfectionism [37,68] and maintain high depression, stress, and anxiety [69], as well as protection factors. The results of this study highlighted the role of self-compassion and concrete rumination in preventing parental burnout. These dimensions not only reduce parental perfectionism [70], but also constitute essential resources to cope with difficulties [71,72], while taking care of oneself and one’s family [43]. Indeed, they are all the more useful when we are going through major difficulties such as those related to the psychosocial and mental health consequences of the pandemic [73]. While parents are subject to significant uncertainties about their own personal lives, their children’s lives, and the future in general, as shown in a study by Li and colleagues [74], concrete ways of thinking and greater caring and understanding of their difficulties would enable them to better cope with these stressors. They could therefore also be targeted in future interventions to prevent and reduce parental burnout. These interventions could be based on mindfulness meditation, which has been shown to be effective in reducing abstract ruminations and increasing self-compassion [34]. Its interventions have also been shown to be effective in reducing depression, anxiety, or insomnia, which one in three to six people in the world’s population seem to be affected by and which the various lockdowns during COVID-19 may have reinforced [75,76]. However, it will be necessary to study specifically the means that help parents practice mindfulness in everyday life while their days are already extremely busy.

## 5. Conclusions

While parental burnout is an increasingly studied problem, research evaluating its determinants and practices to prevent and reduce it is ongoing. The results of this study allow us to begin to identify the protective factors of parental burnout. Among them, mindfulness seems to be an essential determinant, especially because it promotes a more self-compassionate posture and helps to reduce abstract ruminations. However, the limitations of this study invite us to reproduce this research and to continue to identify the processes that mediate the effect of mindfulness on parental burnout. Finally, while the need for targeted interventions is essential to promote mental health in times of Covid-19 [77], the findings of this study promote the implementation of interventions based on mindfulness and self-compassion practices to prevent parental burnout. These interventions could be based on the Mindful Self-Compassion program (MSC) [78] or on Compassionate Mind Training (CMT) [79], two protocols based on mindfulness practices and developing self-compassion.

## Figures and Tables

**Table 1:** Descriptive statistics and correlations (Pearson’s r) among the study variables at Time 1.

Variable	n	M	SD	1	2	3	4	5
1. Trait mindfulness	459	55.8	8.94	—				
2. Parental burnout	459	40.5	34.9	−0.471 *	—			
3. Self-compassion	459	22.4	6.04	0.584 *	−0.462 *	—		
4. Abstract ruminations	459	17.8	4.28	−0.466 *	0.588 *	−0.571 *	—	
5. Concrete ruminations	459	16.4	3.61	0.347 *	−0.503 *	0.477 *	−0.432 *	—

Note. Descriptive statistics and correlations (Pearson’s r) among the study variables at Time 1. M = Mean, SD = Standard deviation, 1 = Trait mindfulness, 2 = Parental burnout, 3 = Self-compassion, 4 = Abstract ruminations, and 5 = Concrete ruminations. * *p* < 0.001. The data were analysed with Jamovi.

**Table 2:** Descriptive statistics and correlations (Pearson’s r) among the study variables at Time 2.

Variable	n	M	SD	1	2	3	4	5
1. Trait mindfulness	459	55.6	8.84	—				
2. Parental burnout	459	35	32.8	−0.525 *	—			
3. Self-compassion	459	22.8	6.18	0.499 *	−0.489 *	—		
4. Abstract ruminations	459	15.7	3.71	−0.491 *	0.567 *	−0.575 *	—	
5. Concrete ruminations	459	18	3.75	0.403 *	−0.445 *	0.484 *	−0.324 *	—

Note. Descriptive statistics and correlations (Pearson’s r) among the study variables at Time 2. M = Mean, SD = Standard deviation, 1 = Trait mindfulness, 2 = Parental burnout, 3 = Self-compassion, 4 = Abstract ruminations, and 5 = Concrete ruminations. * *p* < 0.001. The data were analysed with Jamovi.

## Data Availability

The data supporting the conclusions of this study as well as the Ethics Committee’s advice and the questionnaire are openly available at the Center for Open Science (OSF) at the following address: https://osf.io/64kqd/?view_only=1d7ed9cf76784e2e9cfd523ee7e05333 (accessed on 18 August 2021).
